# Identification of a novel c-Myc inhibitor with antitumor effects on multiple myeloma cells

**DOI:** 10.1042/BSR20181027

**Published:** 2018-09-19

**Authors:** Ruosi Yao, Xiaoyang Sun, Yu Xie, Xiaoshen Sun, Yao Yao, Hujun Li, Zhenyu Li, Jian Gao, Kailin Xu

**Affiliations:** 1 Blood Diseases Institute, Xuzhou Medical University, Xuzhou, Jiangsu, China; 2 Department of Hematology, The Affliated Hospital of Xuzhou Medical University, Xuzhou, Jiangsu, China; 3 Jiangsu Key Laboratory of New Drug Research and Clinical Pharmacy, Xuzhou Medical University, Xuzhou, Jiangsu, China

**Keywords:** apoptosis, high-throughput screening, multiple myeloma, Myc

## Abstract

Increasing evidence shows that c-Myc oncoprotein is tightly associated with multiple myeloma (MM) progression. Herein, we identified compound 7594-0035, which is a novel inhibitor that specifically targets c-Myc. It was identified from the ChemDiv compound database by molecular docking-based, high-throughput virtual screening. Compound 7594-0035 inhibited MM cell proliferation *in vitro*, induced cell cycle G_2_-phase arrest, and triggered MM cell death by disturbing the stability of c-Myc protein. Additionally, we also found that compound 7594-0035 overcame bortezomib (BTZ) drug resistance and increased the killing effect on MM cells in combination with BTZ. The severe combined immune deficiency (SCID) mouse xenograft model revealed that compound 7594-0035 partially decreased the primary tumor growth of Roswell Park Memorial Institute (RPMI)-8226 cells *in vivo*. The novel small molecular compound 7594-0035 described in the present study that targets c-Myc protein is likely to be a promising therapeutic agent for relapsed/refractory MM.

## Introduction

Multiple myeloma (MM) is an extremely dangerous malignancy of bone marrow plasma B cells that accounts for 13% of hematological diseases [1]. In recent years, emerging novel agents have been introduced to treat MM, such as bortezomib (BTZ), carfilzomib, thalidomide, and lenalidomide [2,3]. Despite their great success in frontline treatment, drug resistance has been reported for the clinically used MM therapeutic agents [4]. Therefore, it is urgent to deeply explore the mechanism of MM progression and pursue novel molecular-targetted therapeutic drugs.

As a basic helix–loop–helix leucine zipper protein (bHLH-ZIP) transcription factor, c-Myc has been reported to participate in cell proliferation, cell cycle, differentiation, and apoptosis [5,6]. Additionally, c-Myc is also overexpressed in breast cancer, prostate cancer, lung cancer, colon cancer, lymphoma, and leukemia, and is closely associated with cancer aggressiveness and poor prognosis [7–10]. Many studies have shown that inhibition of c-Myc could lead to cell cycle arrest and apoptosis of tumor cells [11,12]. Certainly, it has also been reported that oncogene c-Myc could be activated in approximately 70% of patient-derived MM cells [13].

Recently, Szabo et al. [14] conducted a retrospective study of 117 patients diagnosed with MM and found that ectopic expression of c-Myc was found in 40% of MM patients, which was closely associated with adverse clinical features and worse survival of MM [15]. Therefore, targetting c-Myc may be a promising therapeutic strategy for MM. Compound 10058-F4, an inhibitor of MYC-MAX heterodimerization, induced apoptosis in primary myeloma clones, but not in U266 MM cells [16]. Unfortunately, 10058-F4 also could not be used *in vivo* due to its rapid degradation [17]. The development of novel drugs for pharmacologic targetting of c-Myc is essential for treatment of relapsed/refractory MM.

Herein, a molecular docking-based, high-throughput virtual screening was carried out to identify potent c-Myc inhibitors. One compound named 7594-0035 stood out because of its high docking score, potent c-Myc inhibitory activity, and significant antitumor effect on MM cells. Our studies identified a novel inhibitor of the c-Myc oncoprotein and suggested that compound 7594-0035 may be a promising therapeutic drug that can be used to target c-Myc in relapsed/refractory MM patients.

## Materials and methods

### High-throughput virtual screening

**Figure 1:**
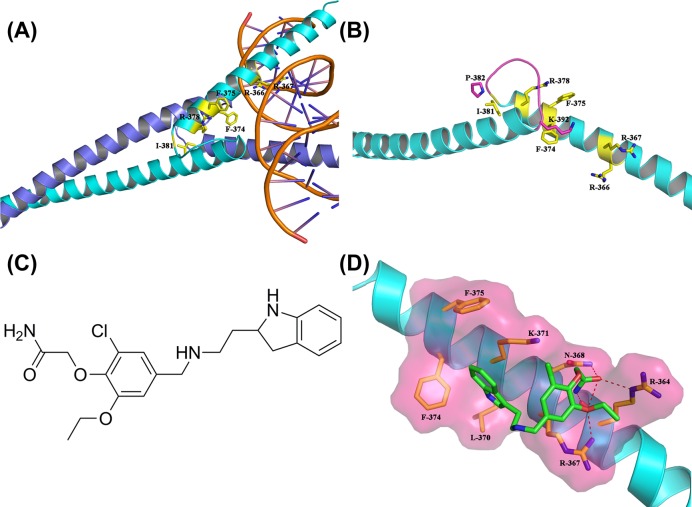
Structure of c-Myc and its potent inhibitor compound 7594-0035 (**A**) Structure of c-Myc-Max recognizing DNA. The key residues for inhibitor binding are shown in stick mode and colored in yellow. (**B**) The detailed inhibitor binding site of c-Myc. The loop^382–392^ (colored in light purple) partly blocked the binding site. (**C**) Structure of compound 7594-0035 obtained from virtual screening. (**D**) Predicted binding of compound 7594-0035 to protein c-Myc, obtained by molecular docking-based virtual screening. The protein c-Myc is shown in cartoon mode and colored in cyan. Compound 7594-0035 is shown in stick mode and colored in green.

The crystal structure of c-Myc-Max recognizing DNA (Protein Data Bank (PDB) ID: 1NKP [18]) for high-throughput virtual screening was obtained from the RCSB (PDB) [19]. The ChemDiv database, a commercially available small molecule database from TopScience Co. (Shanghai, China) containing more than 1 million compounds, was consulted as a screening library. The Surflex molecular docking module in the Sybyl-X2.1 molecular modeling and simulation suite (Tripos Associates, St Louis, MO) was used for high-throughput virtual screening. Because only 2D-structural information was available, all compounds in the ChemDiv database were preprocessed by using the db translate module in Sybyl-X2.1. Considering that there is no ligand in the crystal structure of c-Myc-Max recognizing DNA, the region Arg^363^-Ile^381^ of c-Myc (Figure 1A) was defined as the active site for inhibitor binding, as described in previous molecular docking studies [20]. In the stable state of c-Myc, the loop^382–392^ would close to the active site, especially for Lys^392^, the side chain of which inserts into the active site (Figure 1B). Thus, during the preparation of the receptor, only the region Arg^363^-Ile^381^ was set as the active site, and the loop^382–392^ and all water molecules were removed. To accelerate the virtual screening, a high-speed screening was first carried out by decreasing the maximum quantity of conformations and rotatable bonds from 20 to 10, and from 100 to 50, respectively. Then, the molecules with a docking score within the top 1% were screened again using the default docking parameters. After two rounds of virtual screening, 200 hits were selected by docking score and clustering analysis, and these were commercially purchased for the following biological evaluation.

### Cell culture

Roswell Park Memorial Institute (RPMI)-8226 and U266 cell lines were obtained from the American Type Culture Collection (Manassas, VA, U.S.A.). RPMI-8226/BTZ100 cell lines were kindly provided by Dr Jacqueline Cloos (VU University Medical Center, The Netherlands) [21]. All cells were cultured in RPMI-1640 medium containing 10% FBS at 37°C, 5% CO_2_.

### Cell cycle and proliferation analysis

Briefly, the distribution of the indicated cells in different phases was analyzed by flow cytometry. RPMI-8226 and U266 cells were seeded in six-well plates at approximately 40% density treated with different concentrations of compound 7594-0035. The cell pellets were fixed with cold ethanol and incubated with RNase A. Then, the cells were stained by Propidium Iodide (PI) and examined using an FACSCalibur flow cytometer (BD Biosciences, U.S.A.). For the proliferation assay, the indicated cells were plated in 96-well plates at a density of 1 × 10^4^ per well. The cells were treated with compound 7594-0035 at different concentrations for 48 h or at 30 μM for different amounts of time. Then, cell growth was measured using the Cell Counting Kit-8 (CCK-8) assay.

### Cell apoptosis assay

Cell apoptosis was determined using an Annexin V-FITC/PI Detection Kit, in accordance with the manufacturer’s protocol (KeyGEN, China). The indicated cells were seeded in six-well plates at a density of 30% and were treated with different doses of compound 7594-0035. After 48 h, the cells were stained with Annexin V-FITC and PI and then analyzed by flow cytometry.

### Western blot

The experiments were performed according to a previously described procedure [22]. The following antibodies were used: β-actin (Santa Cruz Biotechnology, CA, U.S.A.), caspase-3 and caspase-9 (Cell Signaling Technology, Beverley, MA, U.S.A.), PARP1, and c-Myc (Proteintech, Chicago, IL, U.S.A.).

### Reverse transcription and Q-PCR

TRIzol reagent (Takara) was used to extract the total RNA from the indicated cells treated with compound 7594-0035 for different amounts of time. Then, 1.5 μg of RNA was reverse transcribed into cDNA according to the manufacturer’s procedure (Promega), and Q-PCR was performed for actin and c-Myc according to a previous report [23].

### Protein stability assay

The indicated cells were treated with 20 μM of compound 7594-0035 for 1 h, and then treated with 5 μg/ml protein synthesis inhibitor cycloheximide (CHX) (VICMED) for 20 and 40 min. Subsequently, the cells were also treated with 20 μM of compound 7594-0035 for 8 h, and then treated with 10 μg/ml proteasome inhibitor MG132 (Selleck) for 3 h. The above protein lysates were used to detect the expression levels of c-Myc protein.

### Xenograft model of MM

Severe combined immune deficiency (SCID) Beige mice (3–4 weeks old) were subcutaneously injected with RPMI-8226 (1 × 10^7^) cells for 1 week. Then, compound 7594-0035 (5 mg/kg body weight, *n*=6) was injected into the tumor every 2 days. Three weeks later, the mice were killed, and tumors were excised. The tumor volume was measured following the formula: *V* = 1/2a × b^2^.

### Statistical analysis

The statistical analysis was performed with Student’s *t* test using GraphPad Prism 5 (GraphPad Software, La Jolla, CA, U.S.A.). *P<0.05 was considered as statistically significant.

## Results and discussion

### High-throughput virtual screening and binding mode of compound 7594-0035

Due to lack of the crystal structure of c-Myc-Max recognizing DNA, the region Arg^363^-Ile^381^ of c-Myc (Figure 1A) was defined as the active site for inhibitor binding, as described in previous molecular docking studies. In the stable state of c-Myc, loop^382–392^ would close to the active site, especially for Lys^392^, the side chain of which inserts into the active site (Figure 1B). Thus, during the preparation of the receptor, only the region Arg^363^-Ile^381^ was set as the active site, and loop^382–392^ and all water molecules were removed. Amongst 200 hits obtained from high-throughput virtual screening, compound 7594-0035 (Figure 1C) showed a high docking score (6.27) and potent c-Myc inhibitory activity (described as follows). To evaluate the binding interaction between compound 7594-0035 and protein c-Myc, the binding mode of compound 7594-0035 was obtained from the virtual screening and is shown in Figure 1D. Compound 7594-0035 formed a strong hydrogen bond network with the side chains of residues Arg^364^, Arg^367^, and Asn^368^, and the main chain of residue Arg^364^. Apart from those polar interactions, the indole ring of compound 7594-0035 was accommodated into a hydrophobic cavity that was formed by Leu^370^, Lys^371^, Phe^374^, and Phe^375^. It is likely that both hydrogen bond interaction and hydrophobic interaction played key roles in the binding of compound 7594-0035 to c-Myc.

### Compound 7594-0035 affects c-Myc protein stability

**Figure 2:**
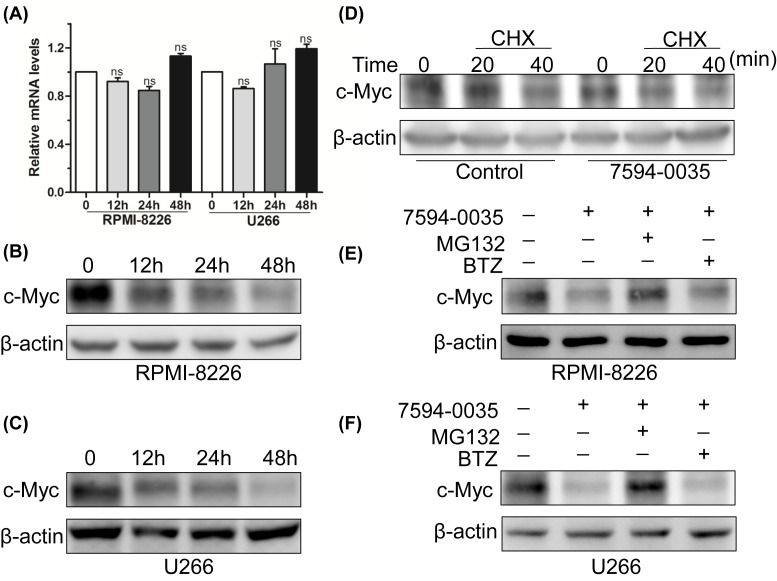
Compound 7594-0035 affects c-Myc protein stability (**A**) Q-PCR analysis of the expression of *c-Myc* mRNA levels in RPMI-8226 and U266 cells treated with 30 μM compound 7594-0035 for different amounts of time. (**B**,**C**) Western blot analysis of the expression of c-Myc protein levels in RPMI-8226 or U266 cells treated with 30 μM compound 7594-0035 for different amounts of time. (**D**) U266 cells were treated with 30 μM compound 7594-0035 for 1 h, and then incubated with CHX for 20 and 40 min. WB detection of the expression of c-Myc protein. (**E**,**F**) Western blot analysis of the c-Myc protein expression in RPMI-8226 or U266 cells treated with compound 7594-0035 for 8 h following incubation with MG132 for 3 h. ns, not significant.

To verify how compound 7594-0035 inhibits c-Myc, we first detected the expression level of *c-Myc* mRNA. The results showed that *c-Myc* mRNA levels were not obviously altered following treatment with 30 μM compound 7594-0035 for different amounts of times in MM cells (Figure 2A). It has been reported that c-Myc is an extremely unstable protein (t_1/2_ = 20–30 min) [24]. Many studies have also proven that c-Myc stability is regulated by cyclin-dependent kinases (CDKs), extracellular signal-regulated kinase (ERK), glycogen synthase kinase 3β (GSK3β), and F-box/WD repeat-containing protein 7 (FBW7) ubiquitin ligase [25–27]. Therefore, we conducted a Western blot assay, and the results demonstrated that compound 7594-0035 decreased the c-Myc protein expression in a time-dependent manner in multiple MM cells (Figure 2B,C), implying that compound 7594-0035 could affect c-Myc oncoprotein stability.

Subsequently, we detected c-Myc protein expression in U266 cells that had been treated with compound 7594-0035 for 1 h, which were then treated with the protein synthesis inhibitor CHX for 20 and 40 min. The results showed that compound 7594-0035 rapidly degraded c-Myc protein after inhibition of protein synthesis (Figure 2D). To further confirm whether compound 7594-0035 affected c-Myc protein stability, RPMI-8226 and U266 cells were treated with compound 7594-0035 for 8 h following treatment with proteasome inhibitor MG132 for 3 h. As expected, compound 7594-0035 caused a significant decrease in c-Myc protein, but this decrease could be reversed by MG132, which could disturb protein degradation (Figure 2E,F). Nevertheless, the proteasome inhibitor BTZ, which represents a breakthrough in the treatment of MM, did not reverse the c-Myc protein expression after treatment with corresponding compound 7594-0035 (Figure 2E,F). The above data further support our findings that c-Myc protein degradation was activated by novel c-Myc inhibitor compound 7594-0035.

### The cytotoxic effect of compound 7594-0035 on MM cells

**Figure 3:**
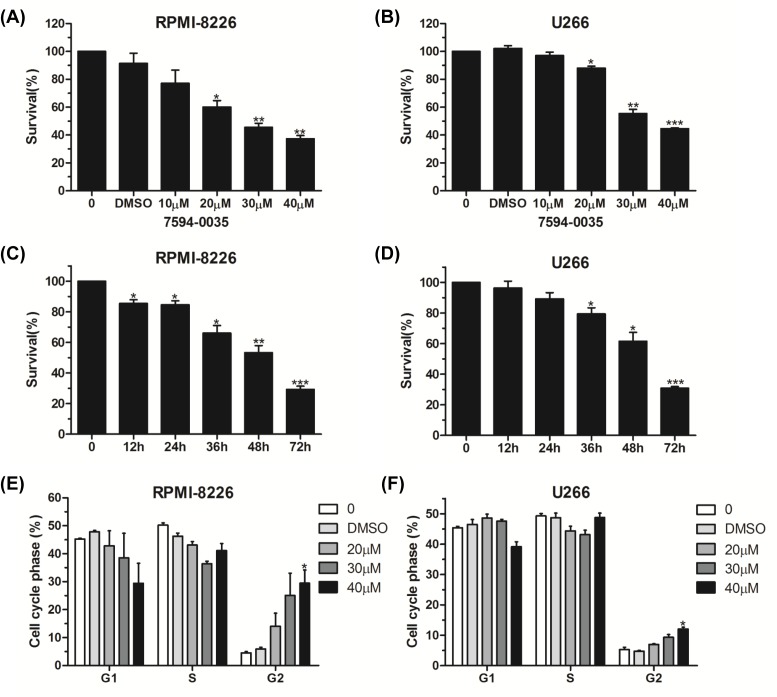
The cytotoxic effect of compound 7594-0035 on MM cells (**A**,**B**) CCK-8 assay analysis of the cell viabilities in RPMI-8226 or U266 cells treated with different doses of compound 7594-0035 for 48 h. (**C**,**D**) RPMI-8226 or U266 cells were treated with 30 μM compound 7594-0035 for different amounts of time and measured with a CCK-8 kit. (**E**,**F**) Flow cytometry analysis of the distribution of the subpopulation in RPMI-8226 and U266 cells after treatment with compound 7594-0035. Error bars, the mean ± S.D.; *, *P*<0.05; **, *P*<0.01; ***, *P*<0.001.

As a novel c-Myc inhibitor, it was necessary to evaluate the cytotoxic effect of compound 7594-0035 on myeloma cells. Both RPMI-8226 and U266 cells were treated with different doses of compound 7594-0035 for 48 h, and the CCK-8 assay confirmed that compound 7594-0035 dose-dependently inhibited MM cell viabilities (Figure 3A,B). As shown in Figure 3C,D, compound 7594-0035 also decreased myeloma cell survival in a time-dependent manner. The dysregulation of cell cycle leading to infinite proliferation is a hallmark of tumor progression [28]. Therefore, we conducted tests to determine whether compound 7594-0035 led to cell cycle arrest. Our flow cytometry assay showed that compound 7594-0035 induced RPMI-8226 and U266 cell cycle G_2_-phase arrest in a time-dependent manner (Figure 3E,F). These data indicate that novel c-Myc inhibitor compound 7594-0035 inhibits MM cell proliferation via mediating cell G_2_-phase arrest.

### The pro-apoptotic effect of compound 7594-0035 on MM cells

**Figure 4:**
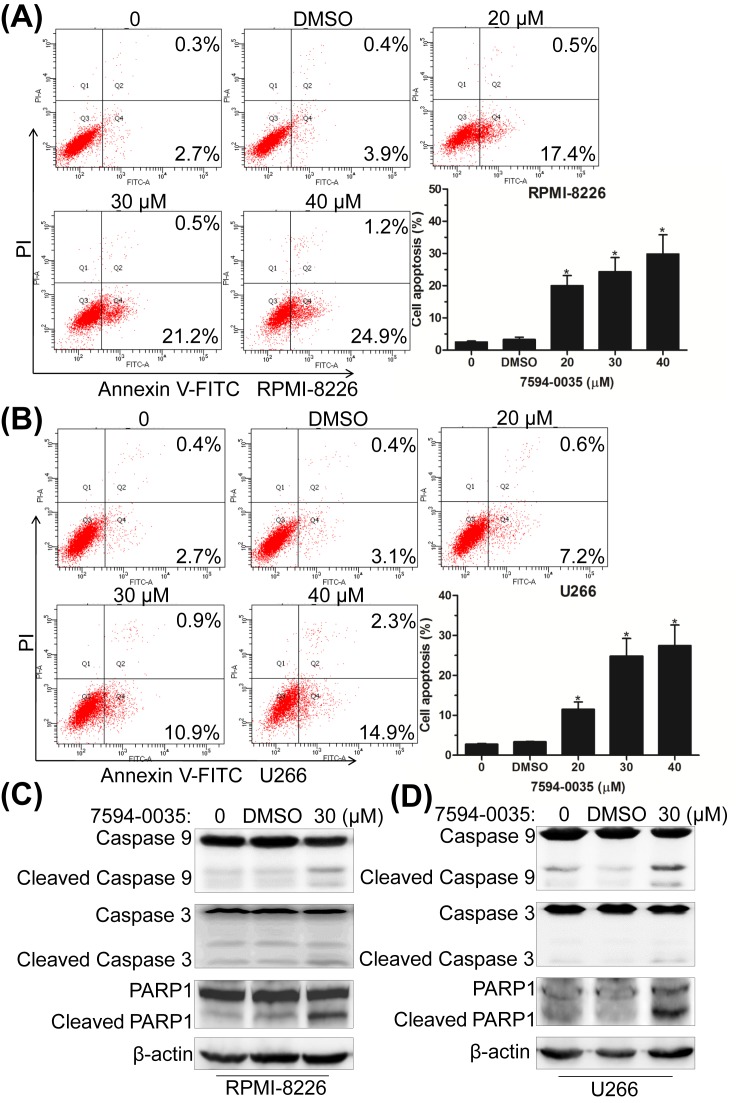
The pro-apoptotic effect of compound 7594-0035 on MM cells (**A**,**B**) Annexin V-FITC/PI staining and flow cytometry analyses of the apoptosis rates in RPMI-8226 and U266 cells treated with different doses of compound 7594-0035. (**C**,**D**) Western blot analyses of the expression of caspase-9, caspase-3, and PARP1 proteins. Error bars, the mean ± S.D.; *, *P*<0.05.

Apoptosis is tightly associated with cell cycle and proliferation [29]. The dysregulation of c-Myc oncoprotein is also important in growth, differentiation, and apoptosis [30]. Herein, we further explored whether compound 7594-0035 had an impact on MM cell death. After treatment with different concentrations of the compound 7594-0035, MM cells were subjected to apoptotic analysis by Annexin V-FITC/PI-labeled flow cytometry. As shown in Figure 4A,B, compound 7594-0035 significantly increased the Annexin V-positive fraction of RPMI-8226 and U266 cells. Additionally, we also detected apoptosis-associated protein expression following treatment with compound 7594-0035 in MM cells. The results showed that caspase-3, caspase-9, and PARP1 were evidently activated (Figure 4C,D), implying that compound 7594-0035 triggered MM cell death via activation of the caspase-related signaling pathway.

### Compound 7594-0035 overcomes drug resistance

**Figure 5:**
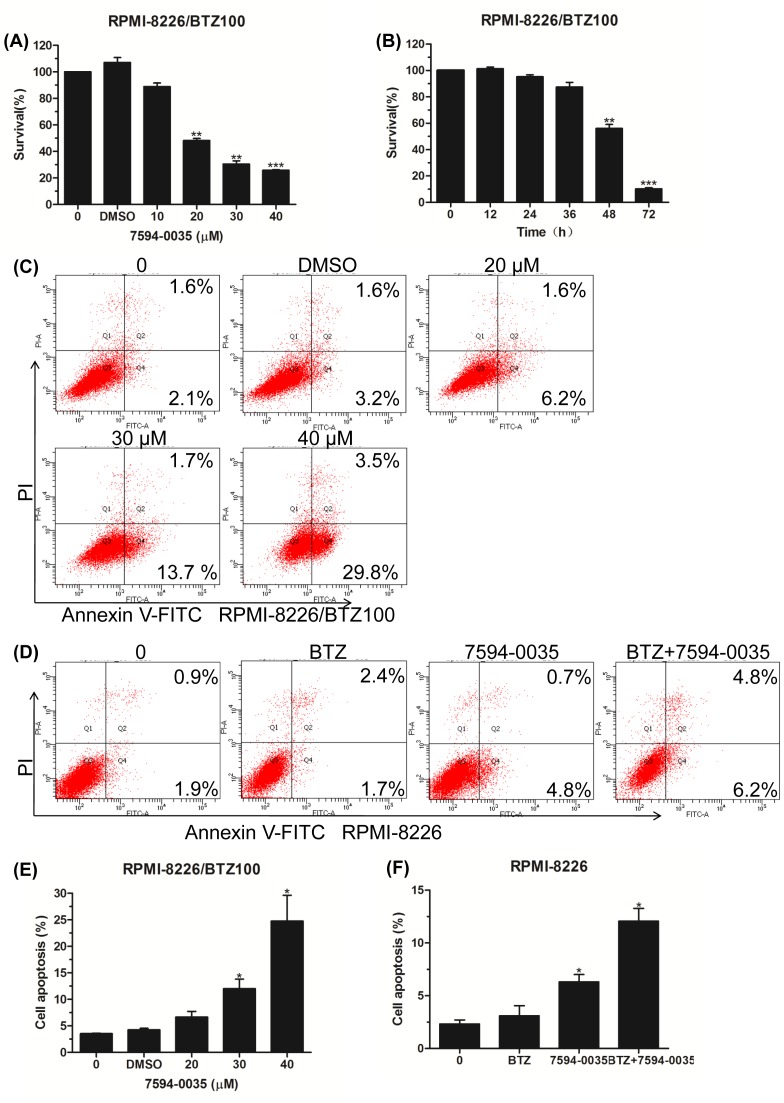
Compound 7594-0035 overcomes drug resistance (**A**) RPMI-8226/BTZ100 cells were treated with different concentrations of compound 7594-0035 and CCK-8 assay analysis shows the cell viability. (**B**) RPMI-8226/BTZ100 cells were treated with 30 μM compound 7594-0035 for different amounts of time, and CCK-8 assay analysis shows the cell viability. (**C**,**E**) Flow cytometry analyses of the apoptosis rates in RPMI-8226/BTZ100 cells. (**D**,**F**) Flow cytometry analyses of the apoptosis rates in RPMI-8226 cells treated with 20 μM compound 7594-0035 and/or 20 nM BTZ. Error bars, the mean ± S.D.; *, *P*<0.05; **, *P*<0.01; ***, *P*<0.001.

As a proteasome inhibitor, BTZ was introduced for treatment of relapsed/refractory MM, which was a great breakthrough as a first-line therapeutic drug [31]. However, in recent years, the phenomenon of BTZ resistance has become more and more prominent. Therefore, exploring the mechanism of drug resistance and developing new therapeutic compounds to overcome resistance are urgent for the therapy of relapsed/refractory MM. In our study, we first detected the cytotoxic effect of compound 7594-0035 on BTZ-resistant MM cell lines. The CCK-8 assay confirmed that compound 7594-0035 inhibited RPMI-8226/BTZ100 cell viabilities in a dose- and time-dependent manner (Figure 5A,B).

Next, we wanted to explore whether compound 7594-0035 could induce apoptosis in BTZ-resistant MM cells. The apoptosis rates were obviously higher in RPMI-8226/BTZ100 cells following treatment with different doses of compound 7594-0035 (Figure 5C,E). We further determined if BTZ exerted a synergistic pro-apoptotic effect with compound 7594-0035. Many preliminary experiments were conducted for determining the optimal combinations between BTZ and compound 7594-0035. Our data indicated that the combination of 20 nM BTZ and 20 μM compound 7594-0035 further increased the apoptosis rates of RPMI-8226 cells (Figure 5D,F). Together, these data confirmed that c-Myc inhibitor compound 7594-0035 overcame BTZ drug resistance and increased the killing effect on MM cells in combination with BTZ.

### Compound 7594-0035 partially decreased the primary tumor growth of MM

**Figure 6:**
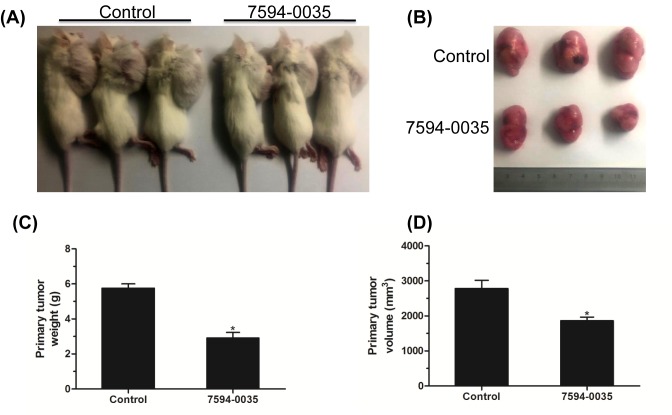
Compound 7594-0035 partially decreases the primary tumor growth of MM (**A**) Representative images of SCID mice carrying RPMI-8226 cells injected with compound 7594-0035 every 2 days. (**B**) Representative images of the primary tumors isolated from xenografted mice. (**C**) Statistical analysis of the weights of the primary tumors. (**D**) Statistical analysis of the volumes of the primary tumors. *P<0.05.

Many small molecular compounds participating in the control of cell growth and induction of apoptosis could be potential antitumor agents [32]. We subcutaneously transplanted RPMI-8226 cells, and after the primary tumor was formed, compound 7594-0035 was injected every 2 days. Three weeks later, the SCID mice were killed, and the primary tumor was isolated (Figure 6A,B). Then, the weights and volumes of tumors were measured, and the statistical analysis indicated that compound 7594-0035 partially reduced the weights and volumes of the RPMI-8226 primary tumors (Figure 6C,D), implying that the monotherapy of compound 7594-0035 is not as effective as combination therapy. Our following work will be focussed on evaluating the effect of the combination of BTZ and compound 7594-0035 on MM primary tumors *in vivo* and further modifying the structure of compound 7594-0035 so as to develop a stronger killing effect on MM cells.

## Conclusion

In the present study, a novel c-Myc inhibitor compound 7594-0035 was identified via high-throughput virtual screening aimed at protein c-Myc. Our studies showed that compound 7594-0035 inhibited the expression of c-Myc protein via disturbing the c-Myc protein stability. With respect to biological functions, we found that compound 7594-0035 decreased the viabilities of MM cells in a dose- and time-dependent manner. Moreover, our data also showed that compound 7594-0035 dose-dependently led to cell cycle G_2_-phase arrest and apoptosis in MM cells. Interestingly, the novel c-Myc inhibitor compound 7594-0035 overcame BTZ drug resistance and increased the killing effect on MM cells in combination with BTZ. *In vivo* xenograft model experiments also demonstrated that compound 7594-0035 partially decreased the primary tumor growth of MM.

## Abbreviations

BTZ: bortezomib
CCK-8: cell counting kit-8
CHX: cycloheximide
MM: multiple myeloma
PDB: Protein Data Bank
PI: propidium iodide
RPMI: Roswell Park Memorial Institute
SCID: severe combined immune deficiency
WD: tryptophan aspartic acid
RCSB: Research Collaboratory for Structural Bioinformatics
PARP1: poly(ADP-ribose) polymerase 1
Q-PCR: quantitative polymerase chain reaction
